# Items

**Published:** 1875-06

**Authors:** 


Centennial Number.—The *Boston Medical and Surgical Journal* for June 17th is a centennial number, and is devoted to historical matters of professional interests. The number is illustrated by a copper-plate portrait of Gen. Joseph Warren, M. D., and opens with a sonnet, by Dr. O. W. Holmes, which contains so many features of beauty that we produce it here :

JOSEPH WARREN.

Trained in the holy art whose lifted shield
Wards off the darts a never-slumbering foe,
By hearth and wayside lurking, waits to throw,
Oppression taught his helpful arm to wield
The slayer's weapon. On the murderous field,
The fiery bolt he challenged laid him low,
Seeking its noblest victim. Even so
The charter of a nation must be sealed !
The healer's brow the hero's honors crowned,
From lowliest duty called to loftiest deed ;
Living, the oak-leaf wreath his temples bound,
Dying, the conqueror's laurel was his meed,
Last on the broken rampart's turf to bleed,
Where freedom's victory in defeat was found.

Following are papers upon the medical profession of Massachusetts at the time of the Revolution, by Dr. George B. Loring ; on the Medical Department of the Continental Army, by Dr. J. M. Toner ; a translation of a Hessian surgeon's notes of his American experiences ; extracts from Gen. Warren's note-book ; reminiscences of a Tory surgeon's part in the battle of Bunker Hill ; and a letter from Charleston, S. C., on historical subjects. The number is a very interesting one, and especially is it so at this time, the centennial of the period it for the most part commemorates.——Change of Dress.—The *Pacific Medical aud Surgical*

*Journal* comes to us in a new dress at the commencement of Volume XVIII. This journal is among our most sprightly exchanges, and we congratulate it upon this evidence of prosperity.——ERRATA.—Dr, George E. Blackham, of Dunkirk, N, Y., writes us that his friend, Lieut. W. L. Carpenter, U. S. A., late of the Hayden Expedition, has called his attention to the substitution of the word *bi*-sexual for *uni*-sexual in his article upon Trichina Spiralis, in our February number. The error occurs upon page 247, fourteenth line from top. The context clearly shows that the mistake was merely a slip of the pen.——The publishers have called our attention to an error in Volume III. of Ziemssens Cyclopædia. On page 290, second text line from the bottom, the word "ounces" should read "drachms," As the mistake might lead to serious consequences, Medical Journals are generally invited to notice it.——NEW JOURNAL.—Dr. Edward B, Stevens, late Ediior of the *Cincinnati Lancet and Obscrver*, announces a new Medical Journal, the *Central New York Jourual of Medicine and Surgery*. The first number will appear in July.——DEATH DURING THE ADMINISTRATION OF ETHER.—Dr. James Hardie, of Manchester, gives in the *British Medical Journal*, the particulars of fatal administration of ether. The patient was a boy aged sixteen, who was suffering from disease of the bones of the foot. On two previous occasions chloroform had been administered, with no unpleasant effects. Before the administration of ether, an ounce of brandy was given to the patient. After inhaling the ether for about four minutes, and upon the application of a fresh amount, the boy suddenly ceased to breathe, and all efforts at re-animation were fruitless.——THE PRESBYTERIAN HOBPITAL.—The trouble between the Lady Manager and certain members of the New York Presbyterian Hospital Staff, seems as far as ever from settlement, and some of our exchanges have contained articles against the connection of ladies with Hospttal Management. We cannot agree with all that has been said in this respect, for we have seen most extellent results in the conduct of the affairs of the Buffalo General Hospital by the Ladies' Hospital Association. Both the Staff and Officers hold them in highest respect. They make it a point, however, not to interfere with the medical affairs in any way.——ITCHING OF THE BONES (?)—Dr. W. P. Armstrong (homoœpathic) advises rhus tox. for "itching of the bones."——SUIT FOR DAMAGES.—From the *Medical and Surgical Reporter* we learn, that two physicians in Quincy, Ill., have been sued by a patient, upon whom they operated for Hernia. The declaration states, that the physicians "conducted themselves in an ignorant manner, by unnecessarily, wantonly, improperly and unskillfully performing a surgical operation on the body of the piaintiff, by cutting through the flesh of said plaintiff into the cavity and through the lower region of the abdomen, and in an unskillful manner the defendants took and removed from the cavity of the abdomen of the plaintiff *twenty-five feet of bowels*, by reason of which ignorance his recovery is greatly impeded." We have not much respect for an attorney who would present, or a court which would entertain such a complaint.——FLORAL.—Among the most interesting non-professional periodicals which come to this office, is the Floral Guide, of Mr. James Vick, of Rochester, N. Y. Mr. Vick is an old editor, and knows how to conduct a work of this kind. We are not versed in the culture of flowers, but those of our friends who are, always take great interest in Vick's Guide. Mr. Vick, by personal attention to his business, has built up a very extensive business in seeds and plants, and his goods may always be relied upon as first-class.

